# Dimensional Accuracy of Novel Vinyl Polysiloxane Compared with Polyether Impression Materials: An In Vitro Study

**DOI:** 10.3390/ma17174221

**Published:** 2024-08-27

**Authors:** Moritz Waldecker, Stefan Rues, Peter Rammelsberg, Wolfgang Bömicke

**Affiliations:** Department of Prosthetic Dentistry, University Hospital Heidelberg, University of Heidelberg, 69120 Heidelberg, Germany; moritz.waldecker@med.uni-heidelberg.de (M.W.); stefan.rues@med.uni-heidelberg.de (S.R.); peter.rammelsberg@med.uni-heidelberg.de (P.R.)

**Keywords:** accuracy, impression technique, vinyl polysiloxane, polyether

## Abstract

Transferring the intraoral situation accurately to the dental laboratory is crucial for fabricating precise restorations. This study aimed to compare the dimensional accuracy of a new hydrophilic quadrofunctional vinyl polysiloxane (VPS) and polyether (PE), in combination with different impression techniques (mono-phase single step or dual-phase single step). The reference model simulated a partially edentulous mandible. Stainless-steel precision balls were welded to specific teeth and were used to detect dimensional deviations. Fifteen impressions were made for each of the following four test groups: (1) VPS mono-phase, (2) PE mono-phase, (3) VPS dual-phase, and (4) PE dual-phase. Global accuracy was measured by deviations from the reference model, while local accuracy focused on the trueness and precision of abutment tooth surfaces. Statistical analysis was conducted using ANOVA (α = 0.05). All distances were underestimated, with the highest global inaccuracies for the cross-arch distance, ranging from −82 µm to −109 µm. The abutment tooth surfaces showed excellent local accuracy for all the materials and techniques, with crown surface trueness < 10 µm and precision < 12 µm. Inlay surfaces had higher inaccuracies (trueness < 15 µm, precision < 26 µm). Within the limitations of this study, all impression materials and techniques can be used to produce models with clinically acceptable accuracy.

## 1. Introduction

The accuracy of fit of tooth-supported restorations depends on many factors during the fabrication process, of which the accuracy of the impression and the resulting cast are probably the most important. The basic prerequisite for accurately fitting dental restorations is an almost error-free transfer of the intraoral situation to the dental laboratory. Today, dentists have two basic approaches to making an impression—the conventional approach using plastic impression materials and the digital approach using an intraoral scan.

Digital impressions are currently the focus of much scientific attention and are being used more and more in everyday practice. Compared with conventional impressions, digital impressions save time [1,2], increase patient comfort [1,2] and, depending on the indication, improve accuracy. However, despite these clinical and economic benefits, there are limitations. One limitation is that subgingival preparation margins, poorly visible proximal contact areas, insufficient mouth opening, or anatomical features in the retromolar space can make digital impressions difficult or even impossible. Another limitation is that intraoral scanners are only feasible for certain indications. While superior fit has been reported for single crowns and three-unit fixed partial dentures fabricated using digital impressions [3], scan volumes larger than half a jaw are considered unsuitable for the fabrication of fixed partial dentures [4,5,6,7]. Finally, for technical or physical reasons, there is currently no straightforward way to fabricate removable partial or complete dentures based on digital impressions alone [8,9]. Therefore, conventional impressions still play an important role in everyday dental practice. 

Two main materials are used to take conventional impressions of teeth or implants supporting fixed or removable dental prosthesis—polyether (PE) and vinyl polysiloxane (VPS) [10,11]. 

There is no ideal material for every situation [12,13], with each material having its own limitations. Therefore, the dental industry continues to develop improved or even novel materials for conventional impressions. However, the suitability of these new materials must be scientifically tested.

The aim of this study was to compare the dimensional accuracy of a novel VPS material with improved hydrophilicity (hydrophilic quadrofunctional vinyl polysiloxane) with that of established PE materials in combination with different impression techniques (mono-phase single step or dual-phase single step) over short and long distances as well as their accuracy (trueness and precision) and angular changes at the abutment tooth level. The null hypotheses were that accuracy would not be influenced by material class, impression technique, or impression material.

## 2. Materials and Methods

**Figure 1:**
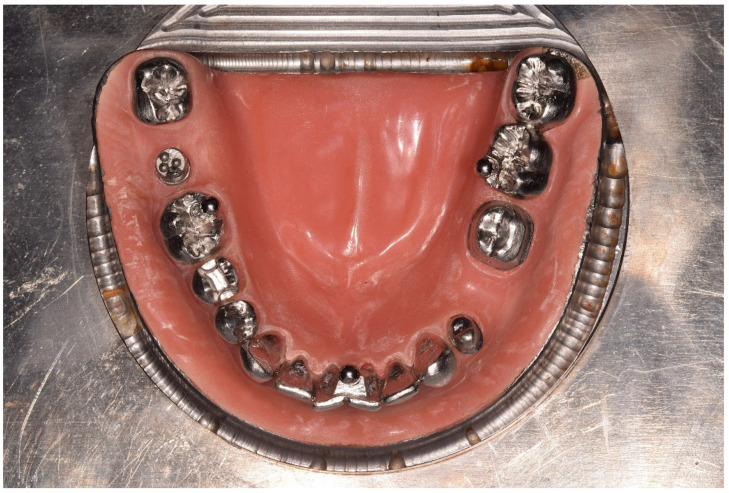
Occlusal view of the reference model.

An edentulous mandibular arch was fabricated from steel and fitted with cobalt–chromium teeth (Figure 1). The partially edentulous arch model simulated the conditions for a fixed partial denture with complete crown preparations on the left first premolar (LP) and first molar (LM) and a trihydral inlay preparation on the right second premolar (RP). Stainless-steel precision balls (diameter = 3.175 mm; G3; shape deviation ≤ 0.08 µm; mean roughness value Ra ≤ 0.01 µm; variation of ball diameter ≤ 0.13 µm) were welded onto the right second molar (B_1_ with center P_1_), onto the left first molar (B_2_ with center P_2_), and between the central incisors (B_3_ with center P_3_). The model was covered with polymethyl methacrylate resin to simulate the attached gingiva.

Before being welded to the model base, all prepared teeth were measured with high precision to create a digital reference dataset on the tooth level (µscan with CF4 sensor, NanoFocus AG (Oberhausen, Germany); surface grid = 50 µm; accuracy < 1 µm). To determine the spatial positioning of the precision balls and the prepared teeth after being welded to the steel base, measurements were made using a coordinate measuring machine (Mar-Vision 222, Hexagon Metrology (Wetzlar, Germany); accuracy < 1–2 µm).

**Figure 2:**
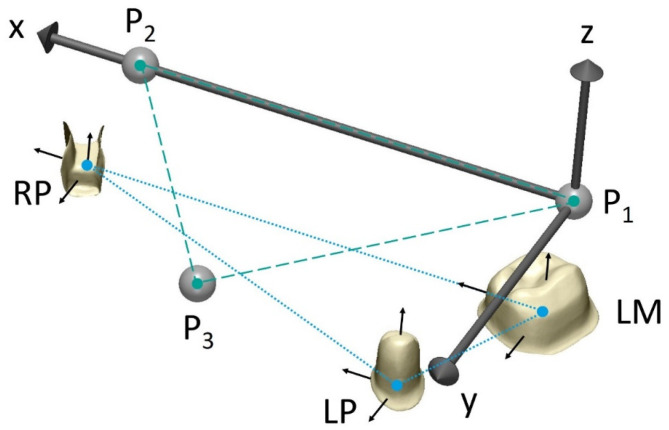
Defined distances between center of each precision ball (turquoise dashed) and between center points on margin level of prepared teeth (blue dotted). LM, left first molar; LP, left first premolar; RP, right first premolar; P_1_, center point of precision ball 1; P_2_, center point of precision ball 2; P_3_, center point of precision ball 3.

A global coordinate system was defined by the centers of the precision balls (P_1_, P_2_, and P_3_) as follows (Figure 2): P_1_ as the origin, x-axis in the direction P_1_P_2_, and the xy-plane defined by all three center points, with the y-axis oriented in the anterior direction. A local coordinate system with axes parallel to those of the global coordinate system was added at the respective center of the margin of each prepared tooth, resulting in angles of 0° between the corresponding tooth axes in the reference model. The reference distances between the center points of the precision balls and of the margins are shown in Table 1. 

A total of 15 impressions per test group were made from the reference model (Table 2). All impressions were removed from the model after 12 min, which is twice the clinical setting time of the PE material and 2.4 times the setting time of the VPS material. The extended setting time at room temperature for VPS was chosen because shrinkage effects could still be detected 10 min (which would be twice the setting time of the material) after mixing. Metallic rim-lock trays were used, individualized with an incisal stop and a dorsal dam, to guarantee a minimum distance between the tray and the metallic reference model and positional stability during setting as well as to support a seamless flow of the impression material to the tooth row. All impressions were disinfected for 5 min (PrintoSept-ID, Alpro Medical GmbH (St. Georgen, Germany)) and then poured with type IV gypsum (esthetic-base gold, dentona AG (Dortmund, Germany)) no earlier than 1 h after removal from the model. The saw-cut models were scanned using a laboratory scanner (D2000, 3shape A/S (Kopenhagen, Denmark)) with a quality control software to generate a digital dataset in the STL file format. 

First, the position of the ball centers (given diameter d = 3.175 mm) was determined by optimization (method of least squares; squared deviations at the triangle corner points were weighted with proportionate surface area using MATLAB version R2020a, MathWorks (Natick, MA, USA), and the deviations for distances defined by the ball centers, ΔP_1_P_2_, ΔP_1_P_3_, and ΔP_2_P_3_, were calculated in relation to the respective reference distances. For the prepared teeth, each reference tooth surface, together with its local coordinate system, was aligned separately to the scan data by means of a best-fit algorithm (Geomagic Design X; 3D Systems (Rock Hill, SC, USA). Distance deviations (ΔLMLP, ΔLMRP, and ΔLPRP) were then assessed between the origins of the coordinate systems (margin level), located at the center of the margin line, and between the intersection points of the vertical axes (z-axes) of the local coordinate systems with the respective occlusal tooth surface (surface level). In addition, angular deviations between the x-axes (Δα), the y-axes (Δβ), and the z-axes (Δγ) after individual tooth alignment were assessed.

Distance deviations were analyzed using both signed and unsigned values. The accuracy of the individual surfaces of the prepared teeth within the margin line was analyzed in terms of trueness (mean mesh deviation between reference and scan) and precision (standard deviation of the mesh deviations along the surface). To evaluate trueness and precision, unsigned (absolute) values were used. 

All results were displayed as boxplot diagrams for descriptive analysis. In boxplots, circles/asterixes mark mild/extreme outliers deviating more than 1.5/3.0 times the interquartile range from the respective quartile value. Significant (α = 0.05) factors were quantified using ANOVA and Tukey’s post hoc tests (SPSS 24 (Armonk, NY, USA)).

## 3. Results

**Figure 3:**
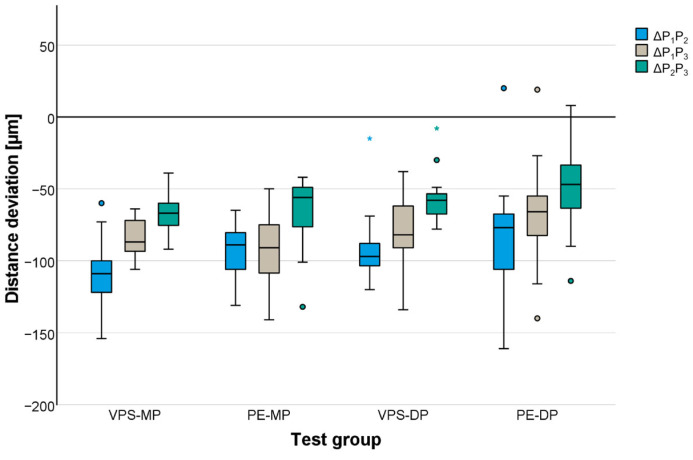
Distance deviations displayed for distances between precision ball center points P_1_, P_2_, and P_3_.

**Figure 4:**
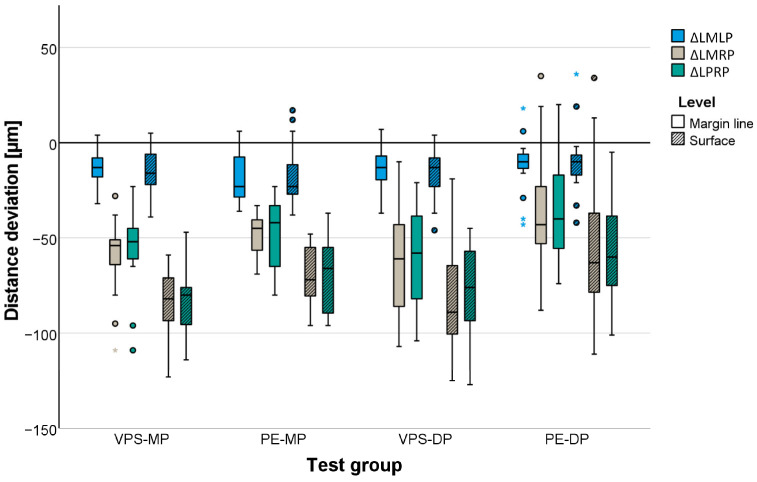
Distance deviations displayed for distances between abutment teeth measured at margin level and at surface level.

In general, distances were underestimated independent of the test group. Mean distance deviations between the precision ball centers were highest for the cross-arch distance (P_1_P_2_) and ranged between −82 μm for the PE-DP group and −109 μm for the VPS-MP group (Table 3, Figure 3). Distances between the abutment teeth (Table 3, Figure 4) were reproduced more accurately. Mean distance deviations were not larger than −63 μm (measured for VPS-DP) at the margin line level and −84 µm (measured for VPS-MP) at the surface level for long distances (LMRP and LPRP). All the gypsum models showed high accuracy independent of the impression material for the frequent clinical application of three-unit fixed partial dentures. The mean deviations for the distance between the respective abutment teeth (LMLP) never exceeded −18 µm (measured for PE-MP) at the margin line level and −16 µm (measured for VPS-MP, PE-MP, and VPS-DP) at the surface level. 

Gypsum casts fabricated from PE impressions showed slightly less deviation from the reference model than those fabricated from the VPS impression materials. The accuracy between the VPS and the PE did not differ more than 15 μm for the mono-phase impressions and 27 μm for the dual-phase impressions. No statistically significant influence of material class was found for distances defined by the precision ball centers (*p* = 0.131), whereas distances between the abutment teeth did have a significant effect (*p* = 0.001). 

For the impression technique (mono-phase/dual-phase), a significant effect was found for both distances defined by the precision ball centers and for distances between the abutment teeth at the surface level (*p* ≤ 0.036) but not for distances between the abutment teeth at the margin line level (*p* = 0.169).

Concerning the distances defined by the precision ball center points, multiple comparisons revealed no significant differences between the two mono-phase impression materials, VPS-MP and PE-MP (*p* = 0.989), and the two dual-phase impression materials, VPS-DP and PE-DP (*p* = 0.265). For distances between the abutment teeth at the margin line and surface level, a significant difference was observed between the dual-phase impression materials (*p* < 0.001) but not for the mono-phase impression materials (*p* ≥ 0.145).

The mean angular deviations between the tooth axes (given by the attached local coordinate systems) ranged between 0.8° and 1.2° for all three axes and all test groups. Maximum angular changes never exceeded 2°. For the most important case, angular changes between the vertical tooth axes (z-axes), the results are given in Table 4. Once again, excellent accuracy was observed for the three-unit fixed partial denture, with mean angular changes of about 0.2° independent of the impression material. For longer distances, the upper limit was 0.8°.

**Figure 5:**
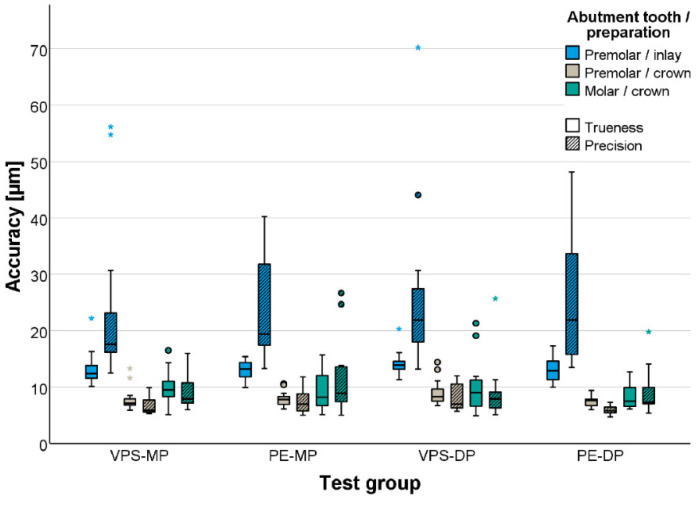
Accuracy (trueness and precision) of prepared teeth.

The local accuracy of the gypsum master casts was comparable in all test groups (*p* = 0.089, Table 5 and Figure 5). Excellent accuracy was obtained for the abutment teeth with full crown preparations (trueness < 10 μm, precision < 12 μm), whereas inlay preparations were more challenging and showed significantly lower accuracy (*p* < 0.001; trueness < 15 μm, precision < 26 μm).

## 4. Discussion

The null hypothesis that accuracy would not be influenced by material class, impression technique, or impression material had to be partially rejected. The local accuracy of the plaster master casts was not different between the groups but did differ between the preparation designs. With respect to global accuracy, the results suggest that the deviations in the plaster master casts generated on the basis of VPS and PE materials were, in general, small but partially significant different. 

This study involved every stage of the workflow, including disinfection of the impression. Disinfection has been shown to influence the impression material’s dimensional accuracy and the surface detail reproduction [14,15]. There is no standard method for disinfecting dental impressions. Various alternative methods can be found in the literature, including the following: ethylene oxide [16], autoclave [16], microwave [17], ultraviolet radiation [18,19], immediate pour and disinfection of the cast [20,21], and chemical disinfection, by the spray or immersion method [22,23]. Chemical disinfection by immersion is considered the most effective method for reducing most microorganisms. As different impression materials have different chemical and physical properties, the manufacturer’s recommendations for disinfection of the respective impression material in terms of duration and method should be strictly adhered to [24,25,26]. Accordingly, this study used a disinfectant in the manner intended by the manufacturer for the impression materials used. Dimensional accuracy and stability of impression materials are crucial factors for successive production of dental restorations. According to Walker et al., regardless of the disinfection protocol (not disinfected or disinfected with two different disinfectant solutions, in combination, with two different time intervals), no significant difference was found for VPS in terms of dimensional accuracy [14]. For PE, a significant difference was found between the disinfected and non-disinfected impressions. This is due to the expansion of PE caused by the absorption of water from the disinfectant solution [14]. In addition to the global accuracy, the local accuracy of an impression is also important. While the surfaces of the VPS impressions showed no changes after disinfection, NaOCL (0.5%) had a significant effect on the surface quality of PE impression, resulting in a mottled or matte/sticky surface. If an impression is not poured directly, the long-term stability of the impression materials is also important. VPS and PE were reported to show a significant difference in their dimensional stability over time. This effect was observed for the non-disinfected and disinfected impressions. However, it is important to emphasize at this point that any changes in accuracy caused by different disinfection methods or registered by measurements at different times have no clinically relevant influence [14,15,22,27,28]. Nevertheless, it seemed reasonable and important to consider disinfection as an integral step in the clinical process of model fabrication, as well as to reflect any potential impact on model accuracy in the results of this study.

Irrespective of the impression material and impression technique, all plaster casts were scaled down overall, and all molded structures experienced a certain degree of lingual tilt. This further shortened the distances at the occlusal surface level compared with distance deviations at the margin level. The direction of scale remains controversial in the literature. Some studies have reported an enlarged scale [29], while others have reported both enlarged and scaled-down measurements [7,30,31]. The reference situation in the present study might have been underestimated because a plaster with less than 0.1% expansion was used, which may not have compensated for the shrinkage of the impression material. Since all distances were too short, a higher expansion of the plaster cast would have improved the accuracy. To compensate the −80 µm to −100 µm deviation on the 40 mm cross-arch distance, a plaster with 0.20% to 0.25% more expansion than the one used in our study would have been necessary. This problem cannot be completely solved in such a simplistic manner since there will be shape deviations that cannot be compensated for by a scaling process, but a higher expansion would have been beneficial in our study since all the distances were underestimated. 

Additionally, the results show that impression accuracy depends on the impression technique, with lower deviations when using the dual-phase technique. This is in contrast to the findings of previous studies. Johnson et al. reported better accuracy with the mono-phase technique than with the dual-phase technique [32], and an earlier study on the same reference model showed that regular-setting polyether impressions performed better with the mono-phase technique than those with the dual-phase technique [33]. Nevertheless, the results are in line with the expectation that the dual-phase technique has greater dimensional stability. Dimensional deviations should be lower with the dual-phase technique because the filler content is higher in the highly viscous phase. 

With regard to local accuracy of the impressions/plaster casts, both trueness and precision were excellent, independent of the material class, impression technique, and impression material. However, the full-crown preparations deviated significantly less than the inlay-preparations, indicating that accurate impressions/plaster casts of inlay preparations are more challenging than those for full-crown-preparations. This might be because of the accessibility of an inlay cavity during the scanning process is much worse than that of a full-crown preparation when digitizing the plaster cast or the more challenging situation during impression-making and pouring of the plaster-casts. However, in general, the observed accuracy of the tested impression materials/techniques is in the range of that which has been demonstrated in previous studies [31,33].

Both impression material class and impression technique are suitable for taking highly accurate impressions of single crowns, fixed partial dentures, inlays, or a complete dental arch. Accuracy differs only slightly between impression materials, so it is reasonable to assume that other factors determine which impression material should be used in a daily routine. This decision may be influenced by economic factors (price, shelf life, storability), patient-related factors (taste, demolding force), physical properties (tear strength, compatibility with astringents or disinfectants, tolerance towards moisture), biological properties (toxicity), and handling differences. Seen in this way, the almost five times higher tear strength of the VPS-DP compared to the PE-DP, in combination with the two to three times easier removal of a VPS impression compared to a PE impression due to less adhesion to the tooth hard substances, might represent a noticeable advantage [13,14]. Impression materials with clinical approval were used in this study. The probability of the impression materials used triggering an allergic or toxic reaction is therefore low. Nevertheless, the literature attributes a certain cytotoxic potential to impression materials [34]. Roberta et al. were able to show that PE drastically reduces cell proliferation in comparison with VPS. At the same time, however, no difference was found between the two materials in terms of their cytotoxicity [35]. A cytotoxic effect was found even after a short exposure time of 10 min of the human gingival fibroblast cells to various impression materials. Dentists should therefore select an impression material with low cytotoxicity and the shortest possible setting time and ensure that all impression residues are removed from the oral cavity [36]. 

As digital impressions cannot currently be used for all indications, it seems reasonable to continue the development of new impression materials and the further development and enhancement of existing impression materials [4,5,6,7,8,9,37]. The focus should be on conventional impression materials that set quickly or whose setting behavior can be controlled by the clinician. 

There are some limitations to this study. This was an in vitro study looking at one partially edentulous situation with prepared and unprepared metal teeth, so the results may not apply to other dental situations. Future studies should be conducted on additional models of different partially edentulous situations. 

That being said, metal teeth do not have uncharacteristically higher demolding forces compared with natural teeth [38]. It can be stated that the material properties of the metal reference teeth in relation to the demolding forces are comparable to the properties of natural teeth. 

Another limitation is that the workflow in this study represents a best-case scenario. The impression tray was fixed and completely immobile during the setting of the impression material, so the clinically relevant influences of patient movements or changes in the position of the impression tray on accuracy were not observed. In addition, the effects of moisture or saliva, sulcus fluid, and blood could not be investigated in vitro.

Moreover, the number of samples is limited. However, 15 samples seem to be a sufficient sample size. In a previous study using an identical model and an identical evaluation strategy, significant differences were found with an even smaller (n = 10) or a similar number of samples (n = 16) [33,38].

## 5. Conclusions

Within the limitations of this study, the following conclusions were drawn:

1. VPS and PE impression materials have adequate accuracy for all clinical applications.
2. The dual-phase impression technique may give a more accurate impression.
3. Short distances are displayed more accurately than long distances regardless of the impression material.
4. Inlay preparations are less accurate than full crown preparations, regardless of the impression material used.
5. The choice of impression material and impression technique lies with the treating clinician and is not only dependent on the accuracy of the impression material.

## Figures and Tables

**Table 1:** Reference distances between center points of precision balls and margins (margin level) as well as between intersection points of vertical axis of local coordinate systems (*z*-axis) with respective tooth surface (surface level).

Distances between Precision Balls			Distances between Prepared Teeth					
[mm]								
P_1_P_2_	P_1_P_3_	P_2_P_3_	LPLM		LMRP		LPRP	
			Margin level	Surface level	Margin level	Surface level	Margin level	Surface level
40.338	35.916	31.927	15.400	15.359	41.515	41.500	36.578	36.498

**Table 2:** Test groups differing in impression material/material combination, material class, and impression technique.

Test Group	Impression Material/Material Combinations	Material Class	Impression Technique
VPS-MP	Aquasil Ultra+ Medium	Vinyl polysiloxane	Mono-phase
PE-MP	Impregum Penta Soft	Polyether	Mono-phase
VPS-DP	Aquasil Ultra+ Heavy/XLV	Vinyl polysiloxane	Dual-phase
PE-DP	Impregum Penta H Duo Soft/Garant L Duo Soft	Polyether	Dual-phase

**Table 3:** Deviations in distances between center points of precision balls and of margins (margin level) as well as between intersection points of vertical axis of local coordinate systems (z-axis) with respective tooth surface (surface level).

Test group	Distance	Level	Distance Deviations [µm]				
			Mean Value	Standard Deviation	Minimum	Median	Maximum
VPS-MP	P_1_P_2_	-	−109	24	−154	−109	−60
	P_1_P_3_	-	−83	14	−106	−87	−64
	P_2_P_3_	-	−66	15	−92	−67	−39
	LMLP	Margin	−13	11	−32	−13	4
		Surface	−16	13	39	−16	5
	LMRP	Margin	−58	21	−109	−53	−28
		Surface	−84	17	−123	−82	−59
	LPRP	Margin	−58	23	−109	−53	−23
		Surface	−84	17	−114	−80	−47
PE-MP	P_1_P_2_	-	−94	20	−131	−89	−65
	P_1_P_3_	-	−93	25	−141	−91	−50
	P_2_P_3_	-	−66	26	−132	−56	−42
	LMLP	Margin	−18	14	−36	−23	6
		Surface	−16	16	−38	−23	17
	LMRP	Margin	−49	12	−69	−45	−33
		Surface	−69	15	−96	−72	−48
	LPRP	Margin	−48	19	−80	−42	−23
		Surface	−69	20	−96	−66	−37
VPS-DP	P_1_P_2_	-	−91	25	−120	−97	−15
	P_1_P_3_	-	−80	27	−134	−82	−38
	P_2_P_3_	-	−56	18	−78	−58	−8
	LMLP	Margin	−13	11	−37	−13	7
		Surface	−16	14	−46	−13	4
	LMRP	Margin	−63	28	−107	−61	−10
		Surface	−83	28	−125	−89	−19
	LPRP	Margin	−59	27	−104	−58	−21
		Surface	−79	26	−127	−76	−45
PE-DP	P_1_P_2_	-	−82	40	−161	−77	20
	P_1_P_3_	-	−68	36	−140	−66	19
	P_2_P_3_	-	−47	32	−114	−47	8
	LMLP	Margin	−12	16	−43	−10	18
		Surface	−9	19	−42	−10	36
	LMRP	Margin	−36	31	−88	−43	35
		Surface	−53	37	−111	−63	34
	LPRP	Margin	−34	29	−74	−40	20
		Surface	−56	29	−101	−60	−5

**Table 4:** Vertical angular deviations for prepared teeth.

Angular Deviation	Test Group	Mean Value	Standard Deviation	Minimum	Median	Maximum
		[°]				
Δα	VPS-MP	1.0	0.1	0.8	1.0	1.3
	PE-MP	1.0	0.2	0.7	1.0	1.8
	VPS-DP	1.0	0.1	0.7	1.0	1.2
	PE-DP	1.1	0.3	0.8	1.1	1.9
Δβ	VPS-MP	1.1	0.1	0.9	1.1	1.2
	PE-MP	1.1	0.2	1.0	1.1	1.7
	VPS-DP	1.0	0.1	0.9	1.0	1.2
	PE-DP	1.2	0.2	1.0	1.2	2.0
Δγ	VPS-MP	0.8	0.1	0.6	0.9	1.0
	PE-MP	0.8	0.1	0.7	0.8	1.1
	VPS-DP	0.8	0.1	0.7	0.8	0.9
	PE-DP	0.9	0.1	0.7	0.8	1.1

**Table 5:** Trueness (precision) for individual prepared teeth.

Tooth	Test Group	Mean Value	Standard Deviation	Minimum	Median	Maximum
		[µm]				
LP	VPS-MP	8 (7)	2 (2)	6 (5)	7 (6)	13 (10)
	PE-MP	8 (7)	1 (2)	6 (5)	8 (7)	11 (12)
	VPS-DP	9 (10)	2 (2)	7 (6)	8 (7)	14 (39)
	PE-DP	7 (6)	1 (1)	6 (5)	8 (6)	9 (7)
LM	VPS-MP	10 (9)	3 (3)	5 (6)	10 (8)	17 (16)
	PE-MP	9 (11)	4 (6)	5 (5)	8 (9)	16 (27)
	VPS-DP	10 (9)	5 (5)	5 (5)	9 (8)	21 (26)
	PE-DP	8 (9)	2 (4)	6 (5)	8 (7)	13 (20)
RP	VPS-MP	13 (23)	3 (14)	10 (13)	12 (18)	22 (56)
	PE-MP	13 (24)	2 (9)	10 (13)	13 (19)	15 (40)
	VPS-DP	14 (26)	2 (14)	11 (13)	14 (22)	20 (70)
	PE-DP	13 (25)	2 (11)	10 (14)	13 (22)	17 (48)

## Data Availability

The original contributions presented in the study are included in the article, further inquiries can be directed to the corresponding author.

## References

[1] Yuzbasioglu E., Kurt H., Turunc R., Bilir H. (2014). Comparison of digital and conventional impression techniques: Evaluation of patients’ perception, treatment comfort, effectiveness and clinical outcomes. BMC Oral Health.

[2] Schepke U., Meijer H.J., Kerdijk W., Cune M.S. (2015). Digital versus analog complete-arch impressions for single-unit premolar implant crowns: Operating time and patient preference. J. Prosthet. Dent..

[3] Chochlidakis K.M., Papaspyridakos P., Geminiani A., Chen C.J., Feng I.J., Ercoli C. (2016). Digital versus conventional impressions for fixed prosthodontics: A systematic review and meta-analysis. J. Prosthet. Dent..

[4] Waldecker M., Rues S., Behnisch R., Rammelsberg P., Bomicke W. (2024). Effect of scan-path length on the scanning accuracy of completely dentate and partially edentulous maxillae. J. Prosthet. Dent..

[5] Waldecker M., Bömicke W., Behnisch R., Rammelsberg P., Rues S. (2021). In-vitro accuracy of complete arch scans of the fully dentate and the partially edentulous maxilla. J. Prosthodont. Res..

[6] Waldecker M., Rues S., Rammelsberg P., Bömicke W. (2020). Accuracy of complete-arch intraoral scans based on confocal microscopy versus optical triangulation: A comparative in vitro study. J. Prosthet. Dent..

[7] Waldecker M., Rues S., Awounvo Awounvo J.S., Rammelsberg P., Bömicke W. (2022). In vitro accuracy of digital and conventional impressions in the partially edentulous maxilla. Clin. Oral Investig..

[8] Cameron A.B., Evans J.L., Robb N.D. (2022). A technical and clinical digital approach to the altered cast technique with an intraoral scanner and polyvinyl siloxane impression material. J. Prosthet. Dent..

[9] Chebib N., Imamura Y., El Osta N., Srinivasan M., Muller F., Maniewicz S. (2022). Fit and retention of complete denture bases: Part II—Conventional impressions versus digital scans: A clinical controlled crossover study. J. Prosthet. Dent..

[10] Hamalian T.A., Nasr E., Chidiac J.J. (2011). Impression materials in fixed prosthodontics: Influence of choice on clinical procedure. J. Prosthodont..

[11] Jayaraman S., Singh B.P., Ramanathan B., Pazhaniappan Pillai M., MacDonald L., Kirubakaran R. (2018). Final-impression techniques and materials for making complete and removable partial dentures. Cochrane Database Syst. Rev..

[12] Guo Y.Q., Ma Y., Cai S.N., Yu H. (2023). Optimal impression materials for implant-supported fixed complete dentures: A systematic review and meta-analysis. J. Prosthet. Dent..

[13] Hüttig F., Klink A., Kohler A., Mutschler M., Rupp F. (2021). Flowability, Tear Strength, and Hydrophilicity of Current Elastomers for Dental Impressions. Materials.

[14] Walker M.P., Rondeau M., Petrie C., Tasca A., Williams K. (2007). Surface quality and long-term dimensional stability of current elastomeric impression materials after disinfection. J. Prosthodont..

[15] Kotsiomiti E., Tzialla A., Hatjivasiliou K. (2008). Accuracy and stability of impression materials subjected to chemical disinfection—A literature review. J. Oral Rehabil..

[16] Holtan J.R., Olin P.S., Rudney J.D. (1991). Dimensional stability of a polyvinylsiloxane impression material following ethylene oxide and steam autoclave sterilization. J. Prosthet. Dent..

[17] Abdelaziz K.M., Hassan A.M., Hodges J.S. (2004). Reproducibility of sterilized rubber impressions. Braz. Dent. J..

[18] Ishida H., Nahara Y., Tamamoto M., Hamada T. (1991). The fungicidal effect of ultraviolet light on impression materials. J. Prosthet. Dent..

[19] Larsen T., Fiehn N.E., Peutzfeldt A., Owall B. (2000). Disinfection of dental impressions and occlusal records by ultraviolet radiation. Eur. J. Prosthodont. Restor. Dent..

[20] Ivanovski S., Savage N.W., Brockhurst P.J., Bird P.S. (1995). Disinfection of dental stone casts: Antimicrobial effects and physical property alterations. Dent. Mater..

[21] Stern M.A., Johnson G.H., Toolson L.B. (1991). An evaluation of dental stones after repeated exposure to spray disinfectants. Part I: Abrasion and compressive strength. J. Prosthet. Dent..

[22] Ahuja B.M., Pawashe K.G., Sanyal P.K., Al-Qarni M.A., Alqahtani N.M., Alqahtani S.M., Ahmed A.R., Abdul Khader M., Elmahdi A.E., Chaturvedi S. (2024). Assessment of dimensional stability of novel VPES impression material at different time intervals with standard disinfectants. BMC Oral Health.

[23] Hardan L., Bourgi R., Cuevas-Suarez C.E., Lukomska-Szymanska M., Cornejo-Rios E., Tosco V., Monterubbianesi R., Mancino S., Eid A., Mancino D. (2022). Disinfection Procedures and Their Effect on the Microorganism Colonization of Dental Impression Materials: A Systematic Review and Meta-Analysis of In Vitro Studies. Bioengineering.

[24] Chidambaram S.R., George A.M., Muralidharan N.P., Prasanna Arvind T.R., Subramanian A., Rahaman F. (2022). Current overview for chemical disinfection of dental impressions and models based on its criteria of usage: A microbiological study. Indian J. Dent. Res..

[25] Vrbova R., Bradna P., Bartos M., Roubickova A. (2020). The effect of disinfectants on the accuracy, quality and surface structure of impression materials and gypsum casts: A comparative study using light microscopy, scanning electron microscopy and micro computed tomography. Dent. Mater. J..

[26] Lepe X., Johnson G.H. (1997). Accuracy of polyether and addition silicone after long-term immersion disinfection. J. Prosthet. Dent..

[27] Awod Bin Hassan S., Ali F.A.A., Ibrahim N.A.L., Heboyan A., Ravinder S.S. (2023). Effect of chemical disinfection on the dimensional stability of polyvinyl ether siloxane impression material: A systemic review and meta-analysis. BMC Oral Health.

[28] Soganci G., Cinar D., Caglar A., Yagiz A. (2018). 3D evaluation of the effect of disinfectants on dimensional accuracy and stability of two elastomeric impression materials. Dent. Mater. J..

[29] Caputi S., Varvara G. (2008). Dimensional accuracy of resultant casts made by a monophase, one-step and two-step, and a novel two-step putty/light-body impression technique: An in vitro study. J. Prosthet. Dent..

[30] Hoods-Moonsammy V.J., Owen P., Howes D.G. (2014). A comparison of the accuracy of polyether, polyvinyl siloxane, and plaster impressions for long-span implant-supported prostheses. Int. J. Prosthodont..

[31] Stober T., Johnson G.H., Schmitter M. (2010). Accuracy of the newly formulated vinyl siloxanether elastomeric impression material. J. Prosthet. Dent..

[32] Johnson G.H., Lepe X., Aw T.C. (2003). The effect of surface moisture on detail reproduction of elastomeric impressions. J. Prosthet. Dent..

[33] Zenthöfer A., Rues S., Rammelsberg P., Ruckes D., Stober T. (2020). Accuracy of a New Fast-Setting Polyether Impression Material. Int. J. Prosthodont..

[34] Sydiskis R.J., Gerhardt D.E. (1993). Cytotoxicity of impression materials. J. Prosthet. Dent..

[35] Roberta T., Federico M., Federica B., Antonietta C.M., Sergio B., Ugo C. (2003). Study of the potential cytotoxicity of dental impression materials. Toxicol. Vitro..

[36] Chen S.Y., Chen C.C., Kuo H.W. (2002). Cytotoxicity of dental impression materials. Bull. Environ. Contam. Toxicol..

[37] Boehm S., Rues S., Balzer A., Rammelsberg P., Waldecker M. (2024). Effect of a calibration aid and the intraoral scanner on the registration of a partially edentulous maxilla: An in vitro study. J. Prosthet. Dent..

[38] Rues S., Stober T., Bargum T., Rammelsberg P., Zenthöfer A. (2021). Disposable plastic trays and their effect on polyether and vinyl polysiloxane impression accuracy-an in vitro study. Clin. Oral Investig..

